# Cortisol Patterns Are Associated with T Cell Activation in HIV

**DOI:** 10.1371/journal.pone.0063429

**Published:** 2013-07-26

**Authors:** Sarah Patterson, Patricia Moran, Elissa Epel, Elizabeth Sinclair, Margaret E. Kemeny, Steven G. Deeks, Peter Bacchetti, Michael Acree, Lorrie Epling, Clemens Kirschbaum, Frederick M. Hecht

**Affiliations:** 1 Department of Medicine, University of California San Francisco, San Francisco, California, United States of America; 2 Department of Psychiatry, University of California San Francisco, San Francisco, California, United States of America; 3 Department of Epidemiology and Biostatistics, University of California San Francisco, San Francisco, California, United States of America; 4 Technical University of Dresden, Dresden, Germany; Rush University, United States of America

## Abstract

**Objective:**

The level of T cell activation in untreated HIV disease is strongly and independently associated with risk of immunologic and clinical progression. The factors that influence the level of activation, however, are not fully defined. Since endogenous glucocorticoids are important in regulating inflammation, we sought to determine whether less optimal diurnal cortisol patterns are associated with greater T cell activation.

**Methods:**

We studied 128 HIV-infected adults who were not on treatment and had a CD4^+^ T cell count above 250 cells/µl. We assessed T cell activation by CD38 expression using flow cytometry, and diurnal cortisol was assessed with salivary measurements.

**Results:**

Lower waking cortisol levels correlated with greater T cell immune activation, measured by CD38 mean fluorescent intensity, on CD4^+^ T cells (r = −0.26, p = 0.006). Participants with lower waking cortisol also showed a trend toward greater activation on CD8^+^ T cells (r = −0.17, p = 0.08). A greater diurnal decline in cortisol, usually considered a healthy pattern, correlated with less CD4^+^ (r = 0.24, p = 0.018) and CD8^+^ (r = 0.24, p = 0.017) activation.

**Conclusions:**

These data suggest that the hypothalamic-pituitary-adrenal (HPA) axis contributes to the regulation of T cell activation in HIV. This may represent an important pathway through which psychological states and the HPA axis influence progression of HIV.

## Introduction

Persistently elevated T-cell activation is now recognized as one of the key pathogenic features of HIV infection. T cell activation increases cell turnover, and the high level of T cell activation is thought to be one of the factors leading to depletion of CD4+ T cells [1]. Although both CD4+ and CD8+ T cell activation are associated with disease progression, the association, somewhat paradoxically, is strongest with CD8+ T cells. One possible explanation for this finding is that activated CD4+ T cells undergo apoptosis more rapidly, whereas activated CD8+ T cells tend persist much longer, making CD8+ T cells a better marker of the overall state of T cell activation [1]. After controlling for viral load, the level of CD8^+^ T cell activation in untreated individuals is independently associated with risk of disease progression, as defined by rate of CD4^+^ T cell loss [2] and risk of developing an AIDS defining complication or dying [3]–[6].

The factors influencing T cell activation in HIV infection have not been fully identified. While some factors that may contribute to the level of activation have been identified, such as infection with co-pathogens and microbial translocation through damaged gut mucosa [7], they do not fully explain the observed variability in T cell activation between HIV-infected individuals.

The hypothalamic-pituitary-adrenal (HPA) axis regulates secretion of glucocorticoids, endogenous hormones with potent anti-inflammatory properties. While increased secretion of glucocorticoids in periods of acute stress may prevent excessive immune activation chronic stress, may lead to decreased glucocorticoid sensitivity and impairment in the ability of the HPA axis to regulate the immune system [8]. We hypothesized that relative dysregulation of the HPA axis, as measured by altered patterns of diurnal glucocorticoid secretion, results in greater levels of T cell activation in persons with HIV.

Normal HPA physiology results in a diurnal rhythm of cortisol secretion characterized by a peak shortly after waking, a gradual decline over the course of the day, and a nadir one hour after the usual time of sleep [9]. This rhythm is often altered in persons exposed to chronically stressful situations, resulting in elevated bedtime cortisol [10] and flatter diurnal slopes [10]–[13]. Flatter diurnal cortisol slopes have also been reported to be associated with depressive symptoms in population based studies [14]. Additionally, several studies show associations between severe chronic psychological stress and low morning cortisol [15]–[18].

A potential role of the HPA axis in HIV pathogenesis is supported by emerging observations linking stress and mood with disease outcome in lentiviral infections. For example, severe stressful life events, lack of social support, and chronic depressive symptoms are associated with accelerated progression of HIV [19], [20]. Of note, in one of these studies, elevated morning cortisol was associated with more rapid disease progression (18). Also, in studies of rhesus monkeys, those inoculated with the simian immunodeficiency virus (SIV) exhibited shorter survival when exposed to stressful circumstances [21], [22].

The association between psychological stress and mood and HIV progression is thought to be mediated by molecular messengers of the HPA axis and autonomic nervous system (ANS), and prior research has identified pathways linking the sympathetic nervous system to HIV pathogenesis [23], [24]. Norepinephrine upregulates expression of CCR5 and CXCR4 receptors on lymphocytes, co-receptors for HIV cell entry, which may enhance HIV replication [25]. Other studies in an SIV model suggest that chronic stress can increase sympathetic nervous system innervation of lymph nodes, which in turn causes increased SIV replication [26], [27].

While there is evidence linking the sympathetic nervous system to accelerated progression of HIV, a clear mechanism linking the HPA axis to disease progression has not been established [23], [24]. We hypothesized that the HPA axis influences HIV disease progression by altering the level of T cell activation. To test this hypothesis, we investigated the relationship between the HPA axis and T cell activation in untreated HIV infected persons with early stage disease. Here, we describe the associations between these factors, and present a model in which impaired function of the HPA axis, as often seen in chronic psychological stress, contributes to abnormal T cell activation and accelerated disease progression.

## Methods

### Ethics Statement

The study was reviewed and approved by the UCSF Committee on Human Research. We obtained written informed consent from all of the participants.

### Study design and participants

We performed a cross-sectional study of the relationship between glucocorticoids and T cell activation in HIV by analyzing baseline data from HIV-infected individuals in the University of California San Francisco Staying Well study. While participants went on to an intervention stage, the data presented here were all obtained prior to any study intervention. Inclusion criteria included (1) HIV-1 seropositivity, (2) CD4^+^ T lymphocyte count >250 cells/µl, (3) plasma HIV-1 RNA>100 copies/ml, (4) antiretroviral therapy naive or untreated for at least four months, and (5) 18 or more years of age. Exclusion criteria included a plan to initiate antiretroviral therapy within 12 months from the time of enrollment and current use or use in the past 6 months of immunomodulator drugs.

Subjects were recruited in the San Francisco area through an extensive recruitment network that included referrals from physicians, community based organizations, other research studies, and self referral generated by word of mouth, advertising, and community outreach. The study received approval from the UCSF Committee on Human Research, and all subjects gave written informed consent.

### Measures

We measured salivary cortisol by providing participants with a home saliva collection kit and instructions to collect three saliva samples each day over three days. In addition to providing a practical means of measuring cortisol while subjects perform normal daily activities, measurements of salivary cortisol more accurately reflect serum free cortisol concentrations than do measurements of serum total cortisol. The salivary cortisol concentration is independent of salivary flow [28], [29]. Participants were instructed to collect the first sample immediately after waking, the second sample at 30 minutes post-waking, and the third sample immediately before bed. To ensure that the 30 minute post-waking sample was collected at the accurate time interval from waking, participants were given a timer to set for 30 minutes upon waking in the morning to serve as a reminder. Participants were also provided with a saliva logbook and instructed to record their waking time, bedtime, and collection times. Detailed instruction in sample collection was provided to participants by trained staff.

Saliva samples were collected by having participants drool into collection tubes, which were frozen and stored at −20 C until analysis. Before biochemical analysis, samples were centrifuged at 3000 rpm for 5 minutes, resulting in a clear supernatant of low viscosity. Free salivary cortisol was analyzed with a commercially available chemi-luminescence-assay (CLIA) with a high sensitivity of 0.16 ng/ml (IBM; Hamburg, Germany).

Blood was drawn between 8 am and noon, in acid citrate dextrose venous vacuum collection tubes. Peripheral blood mononuclear cells (PBMCs) were isolated from whole blood by Ficoll-Hypaque density gradient centrifugation within 6 hours of blood drawing, cryopreserved, and stored at the UCSF Biological Specimen Bank.

Several immunophenotypic markers can be used to quantify T cell activation in vivo, but CD38 expression on T cells is the marker that has shown the best characterized ability to predict disease progression in HIV [4]. CD38 is a multifactorial transmembrane glycoprotein that is upregulated during T cell activation and is associated with increased cell-to-cell adhesion, increased levels of cytokine production, and more rapid CD4^+^ T cell proliferation [2], [30], [31]. The prognostic significance of CD38 expression in the setting of HIV appears to be greatest when measured as the mean density of molecules per cell, rather than the percentage of cells expressing CD38, but the proportion of T cells expressing both CD38 and HLA-DR also provides useful prognostic information [6].

We measured expression of CD38 and HLA-DR on T cells at the UCSF Core Immunology Laboratory using flow cytometry and previously described methods that have been optimized and validated for frozen PBMCs [32]. Frozen PBMCs were rapidly thawed in warm media and counted on a Guava PCA (Millipore) with the Viacount assay (Millipore). Cells were transferred to plates and then washed and labeled with Aqua Amine Reactive Dye (AARD, Invitrogen) to discriminate dead cells. Without additional washing, cells were stained with the following fluorescently-conjugated monoclonal antibodies: CD3-Pacific Blue, CD38-PE, HLA-DR-FITC, CD4-PE CY7, (all from BD Bioscience), and CD8-QDot 605 (Invitrogen). In each experiment a fluorescent-minus one control (FMO) was included for CD38 and HLA-DR to determine the cut off for positive staining. Stained cells were washed, fixed in 0.5% formaldehyde (Polyscience), and held at 4 C until analysis (within 18 hours).

**Figure 1:**
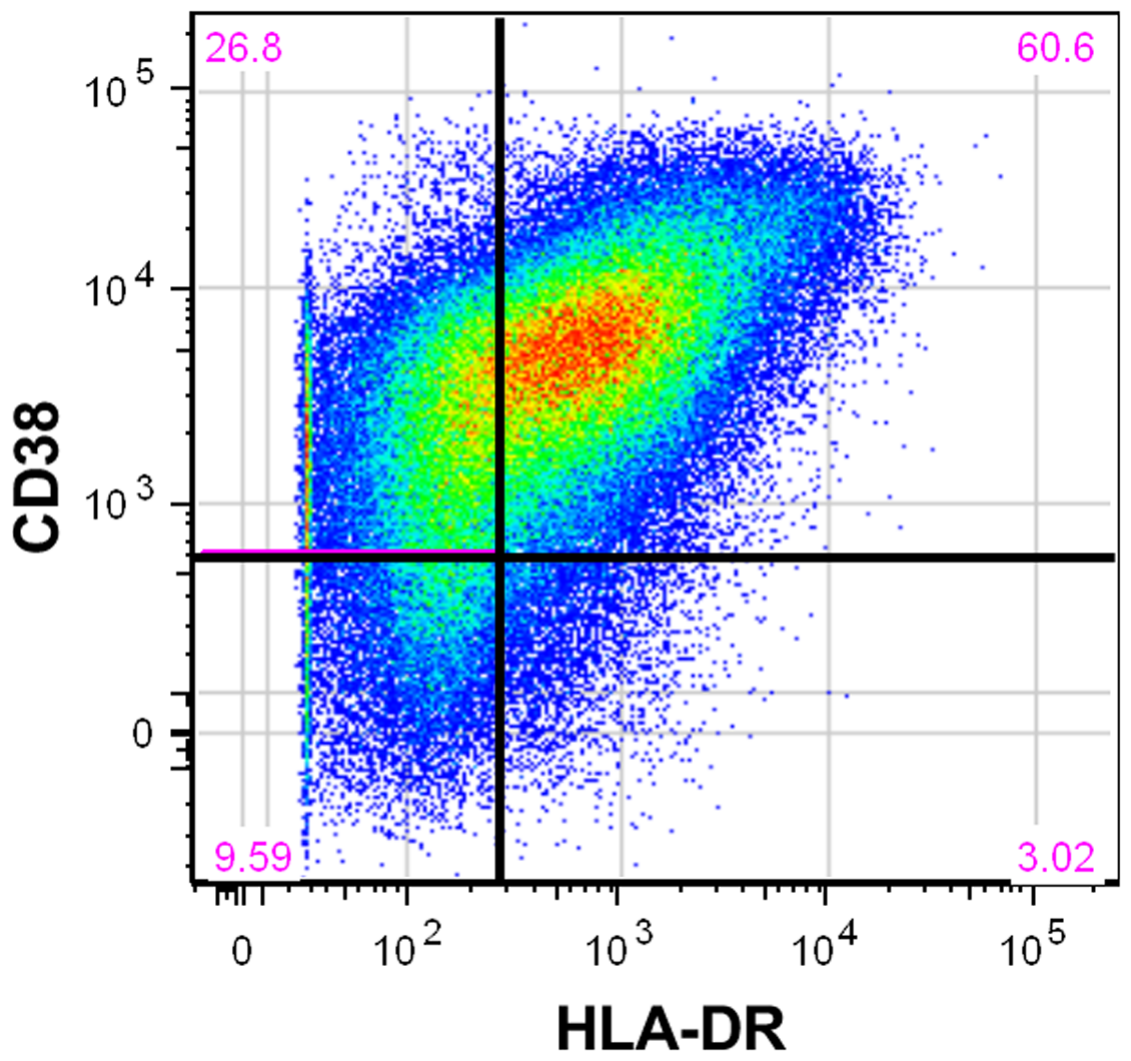
Flow cytometry dot plot of activation markers on CD8+ T cells. Representative dot plot depicting the use of CD38 and HLADR markers to define activated CD8+ T cell populations. The red numbers in each of corner represent the percentage of CD8+ T cells considered to have each combination of the CD38 and HLA-DR markers. For example, 60.6% of CD8+ T-cells in this plot were classified as being positive for both CD38 and HLA-DR. A similar approach was used with CD4+ T cells.

Stained cells were run on a customized BD LSR II flow cytometer and 100,000 to 200,000 lymphocytes were collected for immunophenotyping. Data were compensated and analyzed using Flow Jo (Tree Star) to determine the proportion of CD4^+^ and CD8^+^ T cells expressing each of the T cell markers. Combinations of markers were calculated in FlowJo using the Boolean gate function (see Figure 1 for a typical flow cytometry dot plot that illustrates gating strategy). The geometric mean of relative fluorescence intensity, referred to as the mean fluorescence intensity (MFI), was determined for CD38 on CD4^+^ and CD8^+^ T cells. This approach is similar to the Quantibrite approach [33], but the relative fluorescence units were not converted to the number of molecules per cell as multiple parameters were analyzed simultaneously. Based on previous work showing that CD38 antigen expression on CD8^+^ T cells is the strongest marker of HIV disease progression to AIDS and death, we used CD38 MFI on CD8^+^ as our primary outcome measure [4].

Plasma specimens were used to measure HIV-1 RNA copies per milliliter by polymerase chain reaction using the Amplicor HIV-1 Monitor Test, v1.5 (Roche Diagnostics).

## Analysis

Raw cortisol data were screened for outliers thought to be due to contamination (e.g. blood) or poor participant compliance; samples with values ≥100 nmol/L were deleted. Sample 1 (waking sample) was excluded if collected >10 minutes after waking, sample 2 (30 min post-waking sample) if collected <20 minutes or >45 minutes after waking, and sample 3 (bedtime sample) if collected later than 3 am the following day.

In addition to the cortisol variables representing specific time points across the day, two derived cortisol variables were calculated: the cortisol awakening response (CAR) and cortisol slope. The CAR, or increase in cortisol from waking to 30 min post-waking, was calculated as the difference score between samples 1 and 2 (sample 2 – sample 1). The CAR typically represents a 50% increase in cortisol levels and is larger in persons experiencing chronic worry and stress [34]. The cortisol slope, or degree of change (typically decline) in cortisol levels from early morning to late evening, was computed as the difference between samples 1 and 3 (sample 3 – sample 1). The slope is a global measure of diurnal cortisol rhythmicity; flatter slopes are associated with chronic stress, chronic fatigue, low socioeconomic status, and low occupational status [10]–[13], [16].

Averages were computed for each cortisol variable across the three days of sample collection. Data for cortisol, viral load, and CD4^+^ counts were positively skewed and normalized using a logarithmic (log_10_) transformation. In computing derived variables, raw variables were logarithmically transformed prior to other calculations. Scatterplots were created and pairwise Pearson correlation coefficients calculated to quantify the association between cortisol predictor variables and immunologic outcome variables. Multiple linear regression was used to model T cell activation as a function of diurnal cortisol adjusted for age, viral load, and CD4^+^ count. All analyses were performed using Stata 10.

## Results

Of 177 participants enrolled in the overall study, 128 provided adequate baseline specimens for both cortisol and immunologic assays. Because Pearson correlation coefficients were calculated in a pairwise fashion, and the number of missing datapoints differs across variables, the number of participants used to calculate each coefficient varies within a range of 101–128. The median CD4^+^ T cell count at baseline was 469 cells/mm^3^ (IQR 372–585) and the median plasma HIV RNA level was 4.3 log10 copies/mL (IQR 3.7–4.7) (Table 1).

**Table 1:** Baseline descriptive statistics.

Characteristic	Summary Statistic(s)
Sample size (n)	128
Age, median (10^th^–90^th^ percentile)	40 (29, 55)
Male (%)	96.9
Race/ethnicity (%)	
White	64.1
Black	7.8
Latino	16.4
Other	11.7
CD4 (CD4^+^ cells/mm^3^): median (10^th^–90^th^ percentile)	469 (310, 786)
HIV (log_10_ plasma HIV-1 RNA copies/mL): median (10^th^–90^th^ percentile)	4.3 (3.2, 4.9)
T lymphocyte activation	
CD4 T cell expression of CD38 (MFI^a^): median (10^th^–90^th^ percentile)	954 (583, 1419)
CD8 T cell expression of CD38 (MFI): median (10^th^–90^th^ percentile)	1099 (764, 2181)
HLA-DR^+^ CD38^+^ CD4^+^ T cell %^b^: median (10^th^–90^th^ percentile)	8 (4, 15)
HLA-DR^+^ CD38^+^ CD8^+^ T cell %^c^: median (10^th^–90^th^ percentile)	40 (24, 57)
Diurnal cortisol	
Waking cortisol (nmol/L): median (10^th^–90^th^ percentile)	13 (6, 24)
Waking +30 minute (nmol/L): median (10^th^–90^th^ percentile)	17 (9, 27)
Cortisol awakening response^d^ (nmol/L): median (10^th^–90^th^ percentile)	3 (−5, 13)
Bedtime cortisol (nmol/L): median (10^th^–90^th^ percentile)	3 (1, 14)
Cortisol slope^e^ (nmol/L): median (10^th^–90^th^ percentile)	−10 (−19, −1)

a MFI, or mean fluorescence intensity, is a measure of the density of cell surface antigen.

b Percent of CD4^+^ cells expressing HLA-DR and CD38 cell surface antigens.

c Percent of CD8^+^ cells expressing HLA-DR and CD38 cell surface antigens.

d Cortisol awakening response is the increase in cortisol from waking to 30 minutes post-waking.

e Cortisol slope is the decline in cortisol over the course of the day, from waking to bedtime.

As expected, CD4^+^ count was negatively correlated with activation, as measured by CD38 MFI, on both CD4^+^ (r = −0.18, p = 0.047) and CD8^+^ (r = −0.20, p = 0.029) cells. Also consistent with previous work, there was a positive correlation between plasma HIV RNA levels and both CD4^+^ activation (r = 0.33, p<0.001) and CD8^+^ activation (r = 0.48, p<0.001).

**Figure 2:**
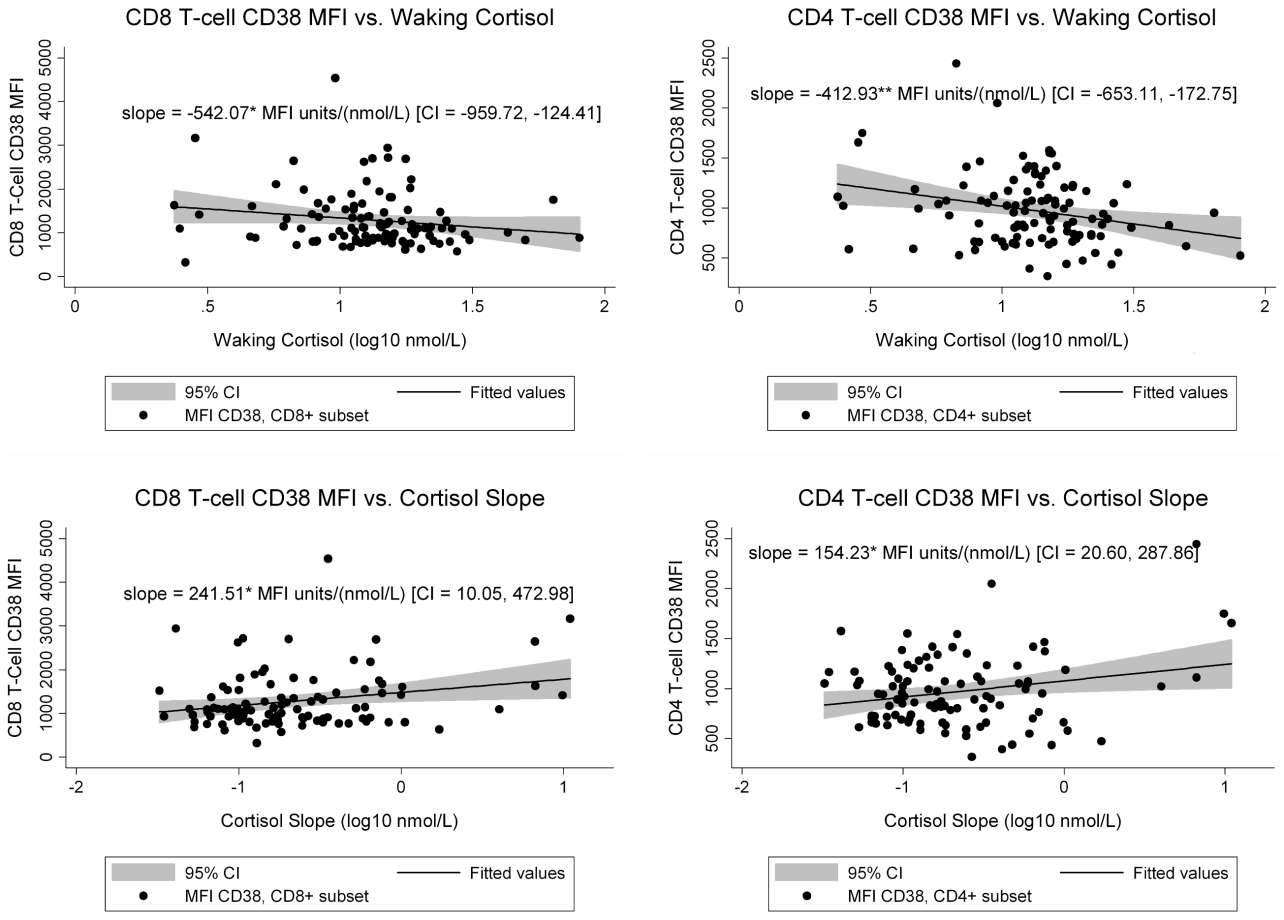
T cell activation as function of diurnal cortisol. The scatter plots show CD8 and CD4 activation as a function of waking cortisol or diurnal cortisol slope. The slope presented in the graphs is the multivariate regression coefficient for T-cell activation as a function of cortisol measures, adjusted for age, viral load, and CD4 count. Figure 2A shows CD38 mean fluorescent intensity (MFI) on CD8+ T cell in relationship to waking cortisol. Figure 2B shows CD38 mean fluorescent intensity (MFI) on CD4+ T cell in relationship to waking cortisol. Figure 2C shows CD38 mean fluorescent intensity (MFI) on CD8+ T cell in relationship to diurnal cortisol slope. Figure 2D shows CD38 mean fluorescent intensity (MFI) on CD4+ T cell in relationship to diurnal cortisol slope. * = p<.05. ** = p≤.001.

We next assessed T cell activation, measured by CD38 MFI, in relationship to cortisol. We found that lower waking cortisol correlated with greater CD4^+^ T cell activation (r = −0.26, p = 0.006) and a trend toward greater CD8^+^ T cell activation (r = −0.17, p = 0.08) (Table 2). In the multivariate regression analysis, waking cortisol was a statistically significant predictor of both CD4^+^ (β = −413 U/nmol/L, p = 0.001) and CD8^+^ activation (β = −542 U/nmol/L, p = 0.011) after adjustment for age, CD4^+^ count, and viral load (Figure 2).

**Table 2:** Correlations of T cell activation measures with cortisol measures^a^.

Cortisol Measure	CD38 on CD8+ T cell MFI	CD38 on CD4+ T cell MFI
Waking cortisol	−0.17 (−0.35, 0.02), p = 0.08	−0.26 (−0.43, −0.08), p = 0.006
30 minute post-waking cortisol	−0.08 (−0.27, 0.11), p = 0.39	−0.20 (−0.37, −0.001), p = 0.049
Cortisol awakening response^b^	0.10 (−0.10, 0.28), p = 0.32	0.11 (−0.09, 0.29), p = 0.28
Bedtime cortisol	0.14 (−0.05, 0.32), p = 0.14	0.07 (−0.12, 0.25), p = 0.50
Cortisol slope^c^	0.24 (0.04, 0.41), p = 0.017	0.24 (0.04, 0.41), p = 0.018

a The values shown are pairwise Pearson correlation coefficients between cortisol measures ((log_10_ nmol/L) and cluster of differentiation (CD) 38 expression on T lymphocytes, measured as mean florescent intensity (MFI), with 95 percent confidence intervals in parentheses, followed by *P*-values for the corresponding correlation coefficients. The N used to calculate the coefficients is 101–109.

b Cortisol awakening response was calculated by subtracting waking cortisol from 30 minute post-waking cortisol, and was typically positive.

c Cortisol slope was calculated by subtracting waking cortisol from bedtime cortisol, and was typically negative.

The 30 minute post-waking cortisol is typically the highest cortisol value over the course of the day. In this study, lower 30 minute post-waking cortisol correlated with greater CD4^+^ activation (r = −0.20, p = 0.049). This relationship was statistically significant following adjustment for age, CD4^+^ count, and viral load (β = −370 U/nmol/L, p = 0.015).

We also examined the relationship between diurnal cortisol slope and T cell activation. Abnormal slope values (less negative or positive) were correlated with greater CD38 MFI on both CD4^+^ (r = 0.24, p = 0.018) and CD8^+^ (r = 0.24, p = 0.017) cells. In addition, cortisol slope was a statistically significant predictor of CD4^+^ and CD8^+^ activation after adjusting for covariates (Figure 2).

Finally, we examined T cell activation as measured by the percentage of T cells that were CD38^+^HLA-DR^+^. Table 3 summarizes the association between cortisol variables and the percentage of CD4^+^/CD8^+^ cells with the CD38^+^HLA-DR^+^ phenotype. Greater bedtime cortisol was correlated with greater HLA-DR^+^CD38^+^CD8^+^%, but this relationship was not statistically significant in the multivariate regression analysis. There was also a non-statistically significant trend toward flatter cortisol slope predicting greater T cell co-expression of HLA-DR/CD38. Overall, we found few associations between diurnal cortisol and percentage of cells co-expressing activation antigens.

**Table 3:** Correlations of T cell percent antigen expression with cortisol measures^a^.

Cortisol Measures	Percent of CD8+ T cells with CD38+HLA-DR+ phenotype	Percent of CD4+ T cells with CD38+HLA-DR+ phenotype
Waking cortisol	0.02 (−0.16, 0.19), p = 0.83	−0.04 (−0.22, 0.13), p = 0.64
30 minute post-waking cortisol	0.003 (−0.17, 0.18), p = 0.98	−0.06 (−0.23, 0.12), p = 0.52
Cortisol awakening response^b^	−0.04 (−0.22, 0.14), p = 0.64	−0.01 (−0.19, 0.17), p = 0.89
Bedtime cortisol	0.21 (0.04, 0.37), p = 0.02	0.15 (−0.02, 0.32), p = 0.09
Cortisol slope^b^	0.16 (−0.02, 0.33), p = 0.09	0.16 (−0.02, 0.33), p = 0.09

a The values presented are pairwise Pearson correlation coefficients between cortisol measures (log_10_ nmol/L) and lymphocyte phenotype, with 95 percent confidence intervals in parentheses, followed by *P*-values for the corresponding correlation coefficients. The N used to calculate the coefficients is 116–128.

b See Table 2 footnotes for explanation of calculated cortisol variables.

## Discussion

Psychosocial stress and mood affect disease outcomes in immune-related disorders such as HIV infection. Although some potential pathways linking these psychological states to disease progression have been identified (23), the mechanisms responsible for this association require further elucidation. Given the strong link between stress, mood, and the neuroendocrine system, we examined whether altered cortisol patterns are associated with greater T cell activation, a known risk factor for accelerated progression of HIV. We found that lower morning cortisol and flatter diurnal rhythms are associated with greater activation of both CD4^+^ and CD8^+^ T cell subsets after adjustment for covariates. To our knowledge, this is the first report of the relationship between diurnal cortisol patterns and immune activation in HIV+ subjects, and the results point to the HPA axis as an important regulator of immune activation in persons with HIV. These findings are particularly significant given the importance of immune activation in the pathogenesis of HIV infection, and imply that improvements in some of the stress-related cortisol patterns could decrease immune activation during HIV infection. Our findings add to an expanding literature supporting a link between stress, the neuroendocrine system, the immune system, and the overall risk of HIV disease progression.

The link between altered cortisol rhythmicity and greater immune activation may be explained by a state in which immune cells demonstrate impaired sensitivity to glucocorticoid signals, or relative glucocorticoid resistance. Studies have shown that chronic psychological stress correlates with impaired immune cell responses to anti-inflammatory signals, including down-regulation of glucocorticoid response elements in leukocytes [35], [36]. These results suggest a model in which chronic stress leads to relative dysregulation of the HPA axis, which in turn causes reduced sensitivity of immune cells to cortisol. Such a loss of sensitivity would impair regulatory responses to dampen inflammation, resulting in higher T cell activation and more rapid progression to advanced disease. Of note, one of the HIV-1 accessory proteins, vpr, has been reported to interact with the glucorticoid receptor and enhance tissue sensitivity to glucocorticoids. This effect might be expected to enhance the effect of normal diurnal variation of cortisol on T-cell activation [37].

Our ability to draw conclusions from this study is limited by its cross-sectional design. While our data show an association between diurnal cortisol and T cell activation, we cannot presently assert a causal relationship between predictor and outcome variables. We suspect that the observed associations are driven by the influence of glucocorticoids on immune function, but it is also conceivable that HPA function is altered in the setting of excessive immune activation. Furthermore, a bidirectional relationship may exist. Data from prospective studies are needed to elucidate the direction of this relationship. In addition, HIV-1 infection itself has also been linked to alterations in the HPA axis [38], which suggests that HIV-1 may be influencing both T-cell activation and the HPA-axis. Prior studies, however, have often reported elevated basal cortisol in advanced infection, or adrenal insufficiency in a subset of patients; our study focused on persons with earlier stage infection and most patients had normal cortisol levels.

Though the statistically significant correlations between diurnal cortisol and immune activation are moderate, they are internally consistent and may be biologically significant. From previous work we know that immune activation in HIV is influenced by multiple factors, including plasma HIV RNA levels, bacterial translocation across altered gut mucosa, age, gender, and infection with co-pathogens such as CMV [1]. We therefore do not expect glucocorticoid levels to account for the majority of variance in activation [2], [39], [40].

We chose to examine CD38 expression due to the evidence supporting its importance in predicting HIV disease progression [3]–[6]. While referred to as an indicator of T-cell activation, it is also important to note that this is a molecule with multiple functions, and not a pure activation marker. In this regard, the relationship that we have shown between cortisol measures and CD38 expression may not represent an association with immune activation alone.

As with most studies of basal cortisol, this study relies on self-report of times when measurements were taken and the relationship between measurement and waking time. To the extent that cortisol patterns truly influence T cell activation, this limitation is likely to cause noise in our predictor variable and underestimation of the true relationship. Some relationships in which we observed trends towards associations, such as increased cortisol awakening response and CD38 MFI on CD4+ and CD8+ T-cells, might not be statistically significant in part due to variability in the collection time of the 30 minute post-waking specimen. We invested considerable effort, however, to minimize errors in the data by screening all cortisol data for participant non-compliance and contacting participants to clarify sampling times when necessary. Given the widths of our confidence intervals and the care with which sampling was performed, we believe the lack of statistical significance in some of the hypothesized relationships does not rule out meaningful associations between particular measures of diurnal cortisol and T cell activation, but suggests that any actual associations are likely to be variable and/or of modest strength.

The most unexpected finding in our study was the apparent difference in the associations between cortisol and our two measures of T cell activation (the density of CD38 on T cells versus the frequency of T cells co-expressing CD38/HLA-DR). There was a consistent association between the density of CD38 on T cells and diurnal cortisol rhythms, but similar associations appeared to be absent (though with some overlap in confidence intervals) between the percentage of cells expressing CD38/HLA-DR and cortisol. This contrast underscores the difference between relative mean fluorescence intensity and percentage measurements for quantifying levels of activation. Glucocorticoids may have more influence on the degree of immune activation than on the percentage of cells in an activated state.

In conclusion, we have shown that cortisol patterns reflective of chronic stress (less diurnal decline, low waking cortisol) were correlated with greater T cell activation in the setting of HIV-1 infection. If supported by further data, these findings indicate a pathway through which psychological states and the HPA axis influence immune function in HIV and other immune-mediated diseases.
